# Effect of recovery time on $\dot{V}{\text{O}}_{2}$-ON kinetics in humans at the onset of moderate-intensity cycling exercise

**DOI:** 10.1007/s00421-022-05057-4

**Published:** 2022-10-17

**Authors:** Silvia Pogliaghi, Enrico Tam, Carlo Capelli

**Affiliations:** 1 Department of Neuroscience, Biomedicine and Movement Sciences, University of Verona, Verona, Italy; 2 Section of Movement Science, Department of Neuroscience, Biomedicine and Movement Sciences, University of Verona, Via Casorati, 43, 37132 Verona, Italy

**Keywords:** Oxygen uptake kinetics, Phosphocreatine, Recovery, Exercise, Oxygen deficit

## Abstract

**Purpose:**

*τ* of the primary phase of $\dot{V}{\text{O}}_{{2{\text{A}}}}$ kinetics during square-wave, moderate-intensity exercise mirrors that of PCr splitting (*τ*PCr). Pre-exercise [PCr] and the absolute variations of PCr (∆[PCr]) occurring during transient have been suggested to control *τ*PCr and, in turn, to modulate $\dot{V}{\text{O}}_{{2{\text{A}}}}$ kinetics. In addition, $\dot{V}{\text{O}}_{{2{\text{A}}}}$ kinetics may be slower when exercise initiates from a raised metabolic level, i.e., from a less-favorable energetic state. We verified the hypothesis that: (i) pre-exercise [PCr], (ii) pre-exercise metabolic rate, or (iii) ∆[PCr] may affect the kinetics of muscular oxidative metabolism and, therefore, *τ*.

**Methods:**

To this aim, seven active males (23.0 yy ± 2.3; 1.76 m ± 0.06, $\dot{V}{\text{O}}_{2\max }$: 3.32 L min^−1^ ± 0.67) performed three repetitions of series consisting of six 6-min step exercise transitions of identical workload interspersed with different times of recovery: 30, 60, 90, 120, 300 s.

**Results:**

Mono-exponential fitting was applied to breath-by-breath $\dot{V}{\text{O}}_{{2{\text{A}}}}$, so that *τ* was determined. *τ* decays as a first-order exponential function of the time of recovery (*τ* = 109.5 × *e*^(−*t*/14.0)^ + 18.9 *r*^2^ = 0.32) and linearly decreased as a function of the estimated pre-exercise [PCr] (*τ* = − 1.07 [PCr] + 44.9, *r*^2^ = 0.513, *P* < 0.01); it was unaffected by the estimated ∆[PCr].

**Conclusions:**

Our results in vivo do not confirm the positive linear relationship between *τ* and pre-exercise [PCr] and ∆[PCr]. Instead, $\dot{V}{\text{O}}_{{2{\text{A}}}}$ kinetics seems to be influenced by the pre-exercise metabolic rate and the altered intramuscular energetic state.

## Introduction

Alveolar oxygen uptake ($\dot{V}{\text{O}}_{{2{\text{A}}}}$) at the onset of constant-load, moderate-intensity exercise attains the steady-state value ($\dot{V}{\text{O}}_{{2{\text{ss}}}}$) following kinetics commonly described as the sum of two mono-exponential responses (Whipp and Ward 1990). The first component (Phase 1, or the cardio-dynamic phase) is related to the rapid increment of pulmonary blood flow (Lador et al. 2006; Loeppke et al. 1981; Yoshida et al. 1993). The second (Phase 2, or primary phase): (i) mirrors the O_2_ uptake response of the muscles ($\dot{V}{\text{O}}_{{2{\text{m}}}}$) (Grassi et al. 1996, 1998), (ii) appears after a time delay of about 15 s, and (iii) is characterized by a time constant (*τ*_2_) of around 35–45 s in moderately fit healthy subjects (Poole and Jones 2005).

The slowness of $\dot{V}{\text{O}}_{{2{\text{m}}}}$ kinetics at the onset of exercise implies that the fraction of ATP resynthesized from phosphocreatine (PCr) breakdown before achieving $\dot{V}{\text{O}}_{{2{\text{ss}}}}$ progressively decreases. The amount of energy derived from the splitting of PCr, the biochemical counterpart of the obligatory O_2_ deficit (DefO_2_) (Ferretti et al. 2022;  di Prampero 1981; Piiper et al. 1968), fills the gap between the energy requirement of muscle contraction and the energy provided by the aerobic pathway, and is linearly related to $\dot{V}{\text{O}}_{{2{\text{ss}}}}$. This phenomenon implies that $\dot{V}{\text{O}}_{{2{\text{m}}}}$ and phosphocreatine concentration ([PCr]) attain the corresponding steady states in a mirror fashion following mono-exponential functions characterized by similar *τ* (McCreary et al. 1996; Rossiter et al. 1999; Whipp et al. 1999).

The factors regulating the kinetics of $\dot{V}{\text{O}}_{{2{\text{m}}}}$ and of PCr splitting have been investigated in detail by applying several experimental approaches, and various models of the regulation of the mitochondrial respiration in skeletal muscles have been explained (Meyer 1988; Grassi 2003; Haseler et al. 2004; Kindig et al. 2005). Korzeniewski and Zoladz, for instance, proposed a dynamical model of the respiratory control in muscle fibers based on the quantitative integration of kinetic equations, features of putative enzymes, and biochemical pathways-blocks (Korzeniewski and Zoladz 2001). According to this model, the *τ* of muscular PCr splitting (*τ*PCr) (and therefore of muscular and alveolar $\dot{V}{\text{O}}_{2}$ kinetics) is related to the decrease of PCr during the rest-to-work transition (∆PCr) in such a way that *τ*PCr increases linearly as a function of ∆PCr (Korzeniewski and Zoladz 2004). The authors, in agreement with their hypothesis, state that “after the termination of an exercise, if [PCr] did not return to its resting level and a new exercise is started, ∆PCr for this principal exercise will, of course, be smaller than for the previous exercise and therefore t1/2 also will be smaller.” (Fig. 5 and page 708, Korzeniewski and Zoladz 2004). In compliance with the aforementioned theoretical model, experimental data showed a correlation between *τ*PCr at the onset of square-wave exercise and ∆PCr (Greiner et al. 2007). However, more recent findings, based on ^31^P-NMR spectroscopy measures of PCr breakdown at the onset of moderate-intensity, square-wave exercise, did not confirm a direct relationship between ∆PCr and the speed of PCr splitting (Francescato et al. 2008). Instead, these data suggested that [PCr] is the primary controller of oxidative phosphorylation in the skeletal muscle (Walsh et al. 2001) and acts as a sort of energy buffer with a capacitance that is proportional to the PCr content (Meyer 1989). Consequently, *τ*PCr is slower (longer in time), the more prominent the [PCr] prevailing before exercise onset.

At the end of the exercise, the energy requirement and the ATP splitting rate diminish immediately. Nevertheless, $\dot{V}{\text{O}}_{{2{\text{A}}}}$ gradually decreases following complex kinetics, usually described as the sum of two mono-exponential decays (Cautero et al. 2005). The rapid component of this kinetics mirrors the rate of PCr resynthesis, and the consumed O_2_ is utilized to repay the DefO_2_ contracted at the onset of the exercise (Piiper and Spiller 1970). The *τ* of this decay (35–40 s) implies that DefO_2_ is completely repaid in about 175–200 s, a time likely sufficient for the full recovery of [PCr]. This recovery time also suggests that if the exercise is resumed before the obligatory DefO_2_ has been fully repaid and PCr stores have not been wholly replenished, we start exercising from a lower [PCr]. Suppose the metabolic requirement, i.e., the $\dot{V}{\text{O}}_{{2{\text{ss}}}}$ elicited by the imposed workload, is unchanged. In that case, we also necessarily imply that the ∆PCr induced by the step transition is lower than that observed when starting from rest. Therefore, the imposition of a square-wave transition at identical workloads, but after different recovery times, might be a simple and effective method to manipulate in a predetermined fashion the pre-transition [PCr] and the ∆PCr of square-wave exercise and to evaluate their effect on the ensuing $\dot{V}{\text{O}}_{{2{\text{A}}}}$ kinetics.

The possible role of an elevated metabolic rate, dissociated from on $\dot{V}{\text{O}}_{{2{\text{A}}}}$ kinetics, has been investigated. In one study, for instance, the investigators showed that the dynamic response of $\dot{V}{\text{O}}_{{2{\text{A}}}}$, and hence of muscular aerobic metabolism, is decelerated when the exercise is resumed starting, during recovery, from a raised metabolic rate (Bowen et al. 2011). This finding was attributed to the recovering muscle's less-favorable energetic status, which directly caused a longer delay of $\dot{V}{\text{O}}_{{2{\text{A}}}}$ in attaining the steady state.

The present investigation was undertaken to challenge these two opposing hypotheses regarding the behavior of $\dot{V}{\text{O}}_{{2{\text{A}}}}$ kinetics when the exercise is resumed after different recovery times. The first one predicts that smaller values of starting [PCr] before the onset of the exercise are related to a progressive acceleration of $\dot{V}{\text{O}}_{{2{\text{A}}}}$ kinetics. The opposite hypothesis states that the raised metabolic rate and the disadvantageous energetic state of the muscle may be associated with a slower $\dot{V}{\text{O}}_{{2{\text{A}}}}$ kinetics. To this aim, we studied in exercising humans the effects of different recovery times on $\dot{V}{\text{O}}_{{2{\text{A}}}}$ kinetics during a square-wave cycling exercise transition performed at the same mechanical power. By manipulating the time of recovery, we induced conditions characterized by metabolic rates and [PCr], which are in opposition: for short recovery times, we have high metabolic rates and low [PCr]; the opposite is true for longer times of recovery. In addition, the results may also allow us to evaluate whether the *τ*_2_ of $\dot{V}{\text{O}}_{{2{\text{A}}}}$ kinetic (from now on, we refer simply to “*τ*”) is linearly related with the estimated ∆PCr occurring during the transients or not.

## Methods

### Subjects

Seven healthy, young men were investigated (age: 23.0 ± 2.3 years; height: 1.76 ± 0.06 m, body mass: 69.8 ± 3.6 kg; $\dot{V}{\text{O}}_{{2{\text{peak}}}}$: 3.32 ± 0.67 L min^−1^; $\dot{V}{\text{O}}_{{2{\text{peak}}}}$ per kg: 47.7 ± 10.7 ml kg^−1^ min^−1^). All the subjects were engaged in regular physical activities. The participants were informed about the procedures and risks associated with the experimental design and protocol, and they signed an informed consent form before participating in the study. The institutional review board of the Department of Neurological and Movement Sciences, University of Verona, approved the study protocol, the experimental design, and methods that conformed to the 1964 Declaration of Helsinki.

### Protocol

The experiments were carried out on the same day of 7 subsequent weeks at the Exercise Physiology Laboratory of the Department of Neuroscience, Biomedicine and Movement Sciences, University of Verona. The subjects were asked to sleep at least 8 h, abstain from vigorous physical activity in the 24 h preceding each test, and consume a light meal 2–3 h before reaching the laboratory. All the tests were performed at approximately the same time of the day under controlled environmental conditions (22–25 °C, 55–65% relative humidity).

On the first day, anthropometric measurements were taken. Then, the subjects were familiarized with the tasks and performed a maximal incremental ramp test to determine $\dot{V}{\text{O}}_{{2{\text{peak}}}}$ and gas-exchange threshold. The cycle ergometer seat and handlebar positions were customized for each subject and then maintained for all subsequent tests in this session.

The ramp test consisted of 3 min at rest, 5 min of warm-up exercise at 50 W, followed by a continuous increase of the workload by 20 W per minute until voluntary exhaustion. The accepted criteria for maximal effort were: respiratory exchange ratio > 1.1 and heart rate (HR) > 90% of the predicted maximum based on age. At the end of the test, blood lactate concentration ([La]_b_) was measured in arterialized capillary blood obtained from an ear lobe (20 μl).

**Fig. 1:**
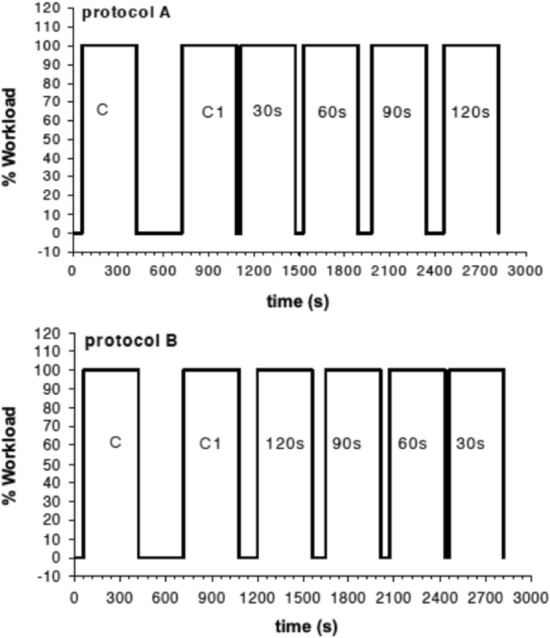
Schematic representation of the applied protocols. In protocol A, after a square transition of 6 min followed by 5 min of recovery, a series of five identical transitions followed. Each exercise phase was separated by increasing times of recovery from 30 to 120 s. In protocol B, the order of the times of recovery was reverted

The constant-load exercise tests were carried out in the following six experimental sessions and consisted of two series of square-wave cycling exercises performed at a workload of 120 W. Each exercise phase lasted 6 min and was interspersed with different times of recovery of 30 s, 60 s, 90 s, 120 s, and 300 s performed at unloaded pedaling maintaining the same RPM used during loaded exercise. Two symmetric sequences (protocol A and protocol B) were devised, and they were administrated randomly to the volunteer in each session to avoid habituation effects (Fig. 1). Each sequence was repeated on different days three times. The first transition was always preceded by a 5-min phase of data collection at rest followed by 3 min of unloaded pedaling. The first constant-load exercise transition (C in Fig. 1) was always followed by a recovery phase of 300 s, during which [La]_b_ was measured at the 1st, 3rd, and 5th min of recovery to assess peak [La]_b_.

### Methods

$\dot{V}{\text{O}}_{{2{\text{A}}}}$ was measured using a metabolic cart (Quark B^2^, Cosmed, Italy). Subjects wore a nose clip and breathed through a mouthpiece mounted on a turbine flow meter. Gases were sampled continuously through a capillary line inserted in the outer frame of the flow meter and analyzed by fast-response O_2_ (chemical) and CO_2_ (infrared) sensors embedded in the metabolic cart. The metabolic cart software allowed us to record gas and flow signals (sampling frequency: 25 Hz) and save them as ASCII files. The analyzers and the propeller were calibrated before each experimental run: (i) with a gas mixture of known composition (FO_2_ = 0.16; FCO_2_ = 0.04; N_2_ as balance) and ambient air; (ii) using a 3 L syringe (Hans Rudolph Inc., USA), following the procedures indicated by the manufacturer. The electromagnetically braked cycle ergometer (SportExcalibur, Lode, D) was connected to and operated by a PC running the metabolic cart. The system made it possible to impose work rates according to pre-defined exercise protocols. The electromechanical characteristics of the ergometer permitted an almost instantaneous step change from one work rate to the next (the response time of the ergometer was around 80 ms, according to the operating manual and as measured in a preliminary session).

[La]_b_, was assessed using an electro-enzymatic method (Biosen C_line, EKF Diagnostics, Barleben, Germany).

*Data analysis.* Single breath alveolar oxygen uptake ($\dot{V}{\text{O}}_{{2{\text{A}}}}$) was then calculated from the original gas and flow traces utilizing an algorithm (Grønlund 1984) that considers variations of the lung’s O_2_ stores that occur from one breath to another. In particular, the N_2_ and Ar concentration was estimated as 100% minus the sum of measured O_2_ and CO_2_ concentrations, as also utilized by Clemensen and colleagues (Clemensen et al. 1994). The algorithm was implemented using an automated procedure written in the object-oriented G language, implemented in the developing environment Labview 5.0 (National Instruments, USA) and running on a PC (HP, USA) (Cautero et al. 2003).

GET was individually estimated by applying the Wasserman method (Wasserman et al. 1999). $\dot{V}{\text{O}}_{{2{\text{peak}}}}$ and all maximal variables were calculated as the average of the last 30 s before the end of the exercise.

The $\dot{V}{\text{O}}_{{2{\text{A}}}}$ time series were aligned with the onset of the first work rate transition of protocols A and B and treated by subtracting the average $\dot{V}{\text{O}}_{{2{\text{A}}}}$ prevailing during the last 15 s preceding the workload change, except for C and C1 where the averages were calculated on the data of the last minute of the phase preceding exercise. Each of the three sequences was then interpolated to a 1-s interval, according to Lamarra et al. (1987), and averaged to get a single data file for each evaluated workload transition in each subject. Finally, each ON-phase of the different square transitions of protocols A and B was separated, plotted as a function of time from 0 to 300 s, and interpolated utilizing a mono-exponential model with a time delay

1 $$Y(t) = A\left( {1 - \exp^{{ - \left( {\left( {t - {\text{TD}}} \right)} \right)/\tau }} } \right),$$

where: *Y*(*t*): $\dot{V}{\text{O}}_{{2{\text{A}}}}$ at time *t*; *A* (L min^−1^) = amplitude of the $\dot{V}{\text{O}}_{{2{\text{A}}}}$ response; TD(*s*) is the time delay of the response; *τ*(*s*) is the time constant of the mono-exponential function.

The parameters of the model [i.e., amplitude (*A*), time constant (*τ*), time delay, TD] were estimated using a weighted non-linear least-squares fitting procedure implemented with Sigmaplot 11 (Systat Software San Jose, CA), as described previously (De Roia et al. 2012). Equation 1 is true only for t > TD and it was fitted without considering the $\dot{V}{\text{O}}_{{2{\text{A}}}}$ data of the first 20 s of each transition. Functional gain (*G*, in ml min^−1^ W^−1^) was calculated as the ratio between $\dot{V}{\text{O}}_{2}$ at steady state and ∆WR, where WR is the work rate.

### Statistics

Values are always presented as mean plus/minus the corresponding standard deviations. Confidence intervals of the estimated *τ* were calculated from the asymptotic standard errors of the parameters (Motulsky and Christopoulos 2004). Differences between the *τ* values obtained from the $\dot{V}{\text{O}}_{{2{\text{A}}}}$-ON kinetics investigated at different recovery times were evaluated using a one-way ANOVA for repeated measures with the recovery time as the within-subjects factor. The sphericity of the pooled data was assessed employing the epsilon statistics. When epsilon was significantly different from 1, the Geisser/Greenhouse correction for the degrees of freedom was applied. Post hoc analysis for the influence of the recovery time on the *τ* of the $\dot{V}{\text{O}}_{{2{\text{A}}}}$-ON kinetics was performed using pairwise comparison using the Tukey multiple comparison test. Linear regressions were calculated utilizing the least-square method, and the corresponding coefficients of determination were obtained. *α* was set to 0.05. The parameters of the non-linear mono-exponential decay of *τ* as a function of recovery time were estimated using weighted non-linear least-squares procedure (Marquardt, 1963). Analysis was performed using the GraphPad Prism version 9.0.1 for macOS (San Diego, CAS, USA).

## Results

The $\dot{V}{\text{O}}_{2}$ corresponding to GET amounted, on average, to 30.5 ± 10.7 ml kg^−1^ min^−1^ and to 47 ± 9.6% of the individual $\dot{V}{\text{O}}_{{2{\text{peak}}}}$. Mechanical power at GET was 159 ± 41. 4 W. Peak [La]_b_ after the C square-wave exercise amounted to 2.7 mM ± 0.07 and peak lactate accumulation was 1.7 mM ± 0.07.

**Fig. 2:**
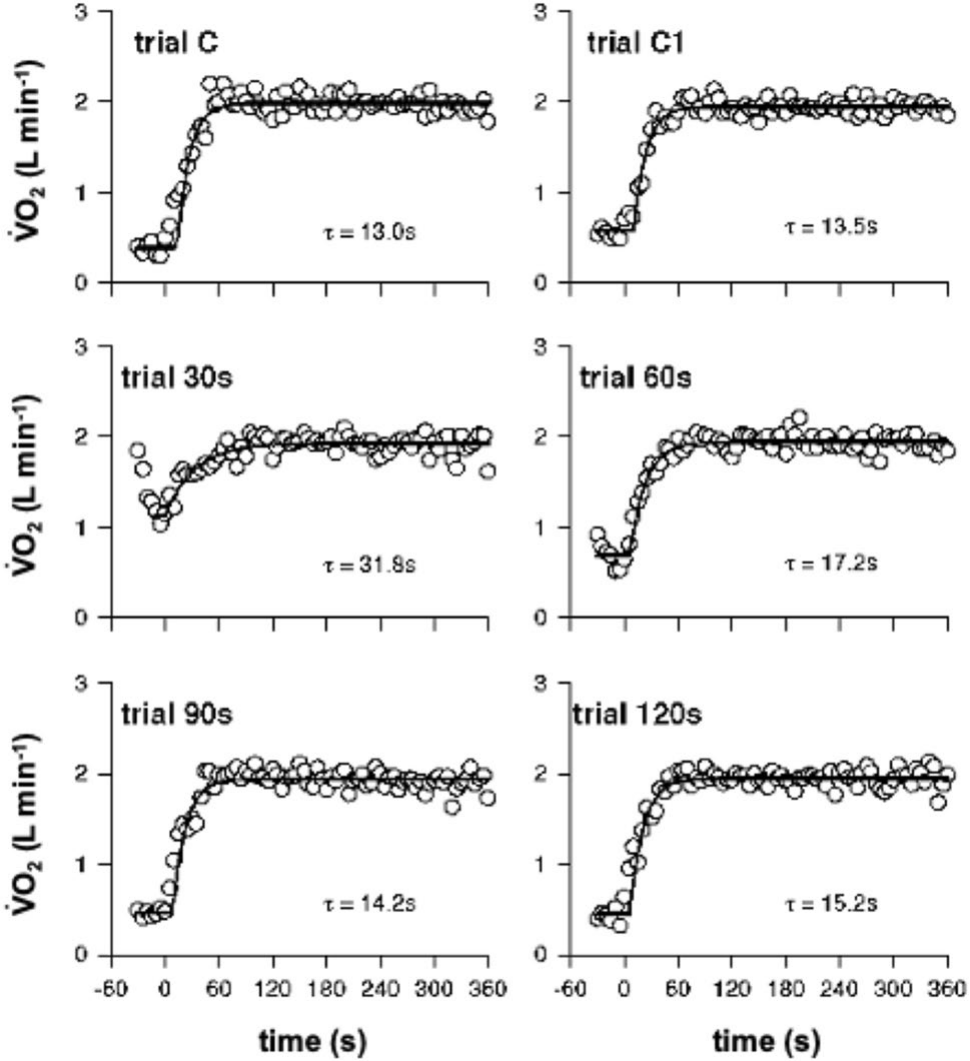
$\dot{V}{\text{O}}_{{2{\text{A}}}}$ kinetics of a typical subjects after interpolation and over-imposition of the single repetitions together with the fitted mono-exponential functions and the resulting *τ*

In Fig. 2, breath-by-breath $\dot{V}{\text{O}}_{{2{\text{A}}}}$ values of a typical subject are reported, after superposition, and synchronization, as a function of time from exercise onset during square-wave transitions performed at the same absolute workload but preceded by variable recovery intervals. The control condition C always started from rest, whereas C1 was preceded by 300 s of recovery. The graphs also report the corresponding mono-exponential fitting functions and the estimated *τ*.

Exercise transitions after the different recovery times started from different metabolic rates. After 30 s of recovery $\dot{V}{\text{O}}_{{2{\text{A}}}}$ was 1.32 l min^−1^ ± 0.13; after 60 s, it amounted to 0.89 l min^−1^ ± 0.13; at 90 s of recovery the subjects resumed exercise when $\dot{V}{\text{O}}_{{2{\text{A}}}}$ corresponded to 0.66 l min^−1^ ± 0.12; and after 120 s, $\dot{V}{\text{O}}_{{2{\text{A}}}}$ was 0.58 l min^−1^ ± 0.95. In addition, $\dot{V}{\text{O}}_{{2{\text{A}}}}$ values prevailing before C (0.44 l min^−1^ ± 0.06) and C1 (0.57 l min^−1^ ± 0.03) were significantly different (*P* < 0.01; 95% CI of difference: 0.10–0.17 l min^−1^).

In Table 1, the average values of the functional gains (*G*), amplitudes (*A*), time constants (*τ*), and time delays (TD) of the $\dot{V}{\text{O}}_{{2{\text{A}}}}$-ON kinetics corresponding to the different recovery times are presented together with their standard deviations and the 95% confidence limits of the estimated *τ*.

**Table 1:** Functional gain (*G*, ml O_2_ min^−1^ W^−1^), amplitude (*A*, l min^−1^), time delay (TD, s), and time constant (*τ*, s) plus 95% limits of confidence of *τ* in the different conditions

Condition	*G*	*A*	TD	*τ*	*τ* 95% limits of confidence
C	11.9 ± 0.9	1.50 ± 0.11	8.2 ± 4.8	23.3 ± 8.1	27.9–19.7
C1	12.0 ± 0.9	1.37 ± 0.18	8.8 ± 5.5	19.0^†^ ± 6.5	23.0–14.9
30 s	12.2 ± 0.7	0.56 ± 0.08	12.6 ± 6.5	28.7 ± 8.0	36.9–20.5
60 s	12.3 ± 0.9	1.09 ± 0.16	8.9 ± 2.5	21.4^*^ ± 5.5	26.8–15.4
90 s	12.2 ± 0.8	1.32 ± 0.13	8.4 ± 4.1	18.6^**^ ± 4.9	23.8–13.4
120 s	12.2 ± 0.8	1.39 ± 0.14	6.7 ± 3.2	19.6 ± 4.4	24.4–14.5

For clarity, only the significant differences among the values of *τ* are reported. For more details, please refer to the text

*Significantly different from 30 s

**Significantly different from 30 s

^†^Significantly different from C

Amplitudes of the responses were significantly different in the various conditions. In detail,

- amplitude of C transition was significantly larger than at C1 (*P* = 0.020), 30 s (*P* < 0.001), 60 s (*P* < 0.001), 90 s (*P* < 0.001), and 120 s (*P* = 0.009);
- amplitude of C1 transition was larger than at 30 s (*P* < 0.001) and 60 s (*P* = 0.003);
- amplitude of 30 s transition was smaller than at 60 s (*P* < 0.001), 90 s (*P* < 0.001), and 120 s (*P* < 0.001);
- amplitude of 60 s transition was smaller than at 90 s (*P* < 0.001) and 120 s (*P* < 0.001);
- amplitude of 90 s transition was smaller (*P* = 0.008) than at 120 s.

Only the amplitudes after 90 s and 120 s of recovery were not significantly different from those assessed after 300 s of recovery (C1).

However, functional gains (grand average 12.1 ml min^−1^ W^−1^ ± 0.15), calculated as the ratio between $\dot{V}{\text{O}}_{{2{\text{A}}}}$ at steady state and workload, and $\dot{V}{\text{O}}_{{2{\text{ss}}}}$ (grand average: 1.96 l min-1 ± 0.02) were not significantly different for the different times of recovery. Time delays were not affected by the recovery time in any of the cases.

**Fig. 3:**
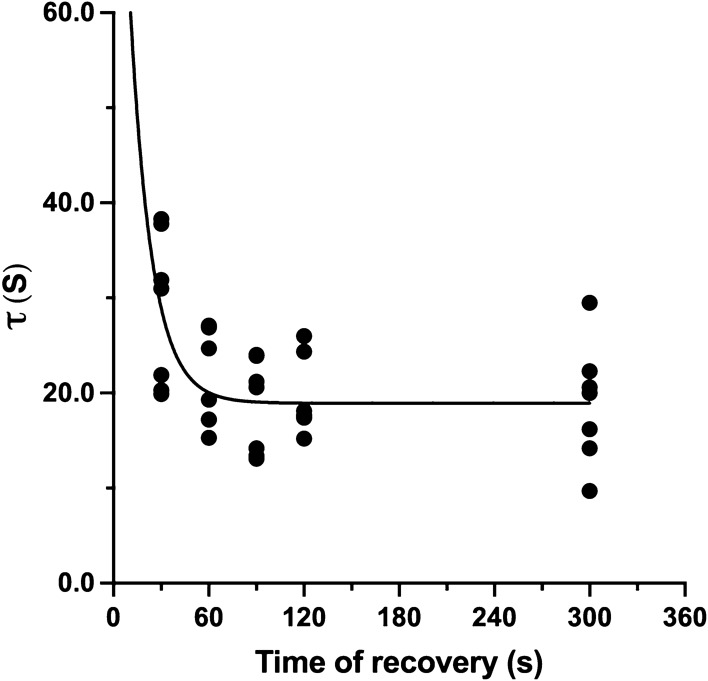
*τ* of $\dot{V}{\text{O}}_{{2{\text{A}}}}$ kinetics as a decreasing function of the recovery time. The decay can be described according to a first-order decaying function

*τ* of the step transition preceded by the shortest time of recovery (30 s) was significantly larger than the ones of the transitions corresponding to longer recovery times (60 s: *P* = 0.035, 95% CI of difference: 0.62–14.6 s; 90 s: *P* = 0.022, 95% CI of difference: 1.67–18.53 s). *τ* of the transition after the shortest time of recovery (30 s) tended to be larger than the one assessed during exercise performed after 120 s of recovery (*P* = 0.0905). Likewise, *τ* assessed after 1 min of recovery tended to be larger than the one estimated during the step transition imposed after 90 s of recovery (*P* = 0.063). Finally, the *τ* of the $\dot{V}{\text{O}}_{{2{\text{A}}}}$-ON response in C was significantly longer than in C1 (*P* = 0.044). The decay of *τ* seemed to decay as a first-order exponential decay of the time of recovery from 30 to 300 s: *τ* = 109.5 × *e*^(−t/14.0)^ + 18.9; *r*^2^ = 0.32) (Fig. 3).

## Discussion

The results obtained in this investigation showed that the *τ* of $\dot{V}{\text{O}}_{{2{\text{A}}}}$-ON kinetics of a square-wave transition imposed after different recovery times at moderate-intensity exercise decreased with recovery time. Since the amount of PCr resynthesized during recovery is proportional to the volume of O_2_ consumed during the rapid phase of the O_2_ debt repayment, this would also imply that *τ* might be affected by the [PCr] existing at the end of recovery or by ∆[PCr] occurring during the subsequent transient. Alternatively, it may be influenced by the raised metabolic rate prevailing before exercise resumption and, hence, on the energetic status of the muscles.

Since *τ* of $\dot{V}{\text{O}}_{{2{\text{A}}}}$-ON kinetics is a close proxy of *τ*PCr, i.e., of the *τ* of PCr splitting at the onset of exercise, we may speculate that the starting [PCr] and/or ∆[PCr] influence, in the same fashion, also the splitting rate of PCr during the transient.

At variance with other investigations where muscular [PCr] and ∆[PCr] were directly assessed during step exercise transitions (f.i., Francescato et al. 2008), in the present study, muscular [PCr] and ∆[PCr] were not directly measured. Therefore, the paragraphs here below are devoted to discussing the current findings in the light of directly obtained data related to the muscular metabolic condition.

### $\dot{V}{\text{O}}_{{2{\text{A}}}}$ kinetics and priming exercise

$\dot{V}{\text{O}}_{{2{\text{A}}}}$ kinetics in the C1 condition, i.e., when exercise was preceded by complete recovery [PCr] after a series of square-wave transitions of moderate-intensity exercise, was significantly faster than when the same workload change was not preceded by any priming exercise (C). In addition, the two amplitudes were significantly different, because C1 initiated from a higher oxygen uptake at rest than in C.

The acceleration of $\dot{V}{\text{O}}_{{2{\text{A}}}}$ kinetics is a common finding (DeLorey et al. 2004; Gerbino et al. 1996; Gurd et al. 2005; Zoladz et al. 2006) when we resume exercise after priming, heavy-intensity exercise. The acceleration of the kinetics has been commonly attributed to better matching between local O_2_ delivery and consumption (De Roia et al. 2012), to a faster response of bulk, cardiovascular O_2_ delivery (Gerbino et al. 1996), or a quick activation of pyruvate dehydrogenase after a bout of exercise (Gurd et al. 2006). In this case, even moderate-intensity exercise elicited a substantial acceleration of the $\dot{V}{\text{O}}_{{2{\text{A}}}}$ kinetics in the subsequent transitions. This unexpected result may be the consequence of a not particularly high level of physical fitness of our volunteers, who, even for a moderate-intensity exercise, could benefit from the priming effect on $\dot{V}{\text{O}}_{{2{\text{A}}}}$ kinetics in the subsequent transition.

### $\dot{V}{\text{O}}_{{2{\text{A}}}}$ kinetics and PCr concentration

As we outlined in the introduction, two models of mitochondrial respiratory control were proposed to explain the link between the splitting rate of muscular PCr during rest-to-work transients and: (i) the split amount of PCr during transient (∆PCr) or (ii) the [PCr] prevailing before the transition.

According to one model, on one hand, the *τ* of $\dot{V}{\text{O}}_{{2{\text{A}}}}$ kinetics is determined by ∆PCr, so that $\dot{V}{\text{O}}_{{2{\text{A}}}}$ kinetics accelerates as ∆PCr decreases (Korzeniewski and Zoladz 2004). On the other hand, data obtained using ^31^P-NMR spectroscopy suggested that the *τ* of PCr splitting during transitions starting from rest is linearly related to resting [PCr] (Francescato et al. 2008). These findings imply that [PCr] itself is one of the main controllers of oxidative phosphorylation in the skeletal muscle (Walsh et al. 2001). According to the investigators, resting [PCr] would act as an energy buffer reserve whose value would be inversely related to the speed of the dynamic response of oxidative metabolism at the beginning of the exercise: the larger the store, the slower the on-kinetics of oxidative metabolism. These findings are also consistent with those obtained in rats fed with a creatine analog that reduced [Cr] and [PCr] contents at rest and with the hypothesis that the creatine kinase reaction acts as a chemical capacitor leading to a value of the *τ* of PCr splitting proportional to the initial [Cr] (Meyer 1989). It is also consistent with a linear model of respiration control in skeletal muscle (Walsh et al. 2001).

Therefore, it is tempting to discuss the present data based on these theories and facts.

In this investigation, muscular [PCr] and ∆[PCr] were not directly measured. Instead, only DefO_2_ could be calculated based on breath-by-breath $\dot{V}{\text{O}}_{{2{\text{A}}}}$ and [PCr] prevailing at the end of the recovery and ∆[PCr] can be therefore tentatively estimated based on: (i) the calculated DefO_2_ and; (ii) some reasonable assumptions concerning the mass of muscles recruited during exercise and muscular [PCr] at rest.

DefO_2_ contracted at the onset of each square-wave transition was first calculated as the difference between: (1) the volume of O_2_ that one should have consumed if $\dot{V}{\text{O}}_{{2{\text{ss}}}}$ had been attained immediately at the beginning of the exercise minus (2) the volume of O_2_ taken up during the exercise. The first member of the difference was calculated by multiplying the difference between (1) $\dot{V}{\text{O}}_{{2{\text{ss}}}}$ and (2) $\dot{V}{\text{O}}_{{2{\text{A}}}}$ at the end of the preceding recovery phase for the duration of the corresponding trial. The second term, i.e., the volume of O_2_ consumed during exercise, was calculated by summing progressively net $\dot{V}{\text{O}}_{{2{\text{A}}}}$ from the onset of exercise to the end of the trial. Afterward, the DefO_2_ in ml of O_2_ was converted in mmol of O_2_ dividing it by 22.4 ml mmol O_2_^−1^. The amount of PCr split during the transient was then calculated by multiplying DefO_2_ (mmol of O_2_) times the P/O_2_ ratio adjusted for the respiratory exchange ratio calculated at steady state (P/O_2_ = 4.13 + 2.07 RER) (di Prampero 1981). To calculate the corresponding ∆[PCr], the absolute value of PCr obtained at the end of the calculations was divided by one-third of the individual body mass, assuming that during cycling, the subjects recruited a muscle mass equivalent to 33% of total body mass. [PCr] at steady state was finally obtained from the difference between [PCr] at rest, assumed equal to 25 mmol kg^−1^ of fresh muscle, and that obtained from DefO_2_. The volume of O_2_ taken up during each recovery phase, the absolute amount of resynthesized PCr, and the corresponding increase of muscular [PCr] were obtained applying similar calculations. Once added to the [PCr] prevailing at steady state, the last value yielded the [PCr] existing at the end of each recovery phase and, hence, before the subsequent square-wave transition.

**Fig. 4:**
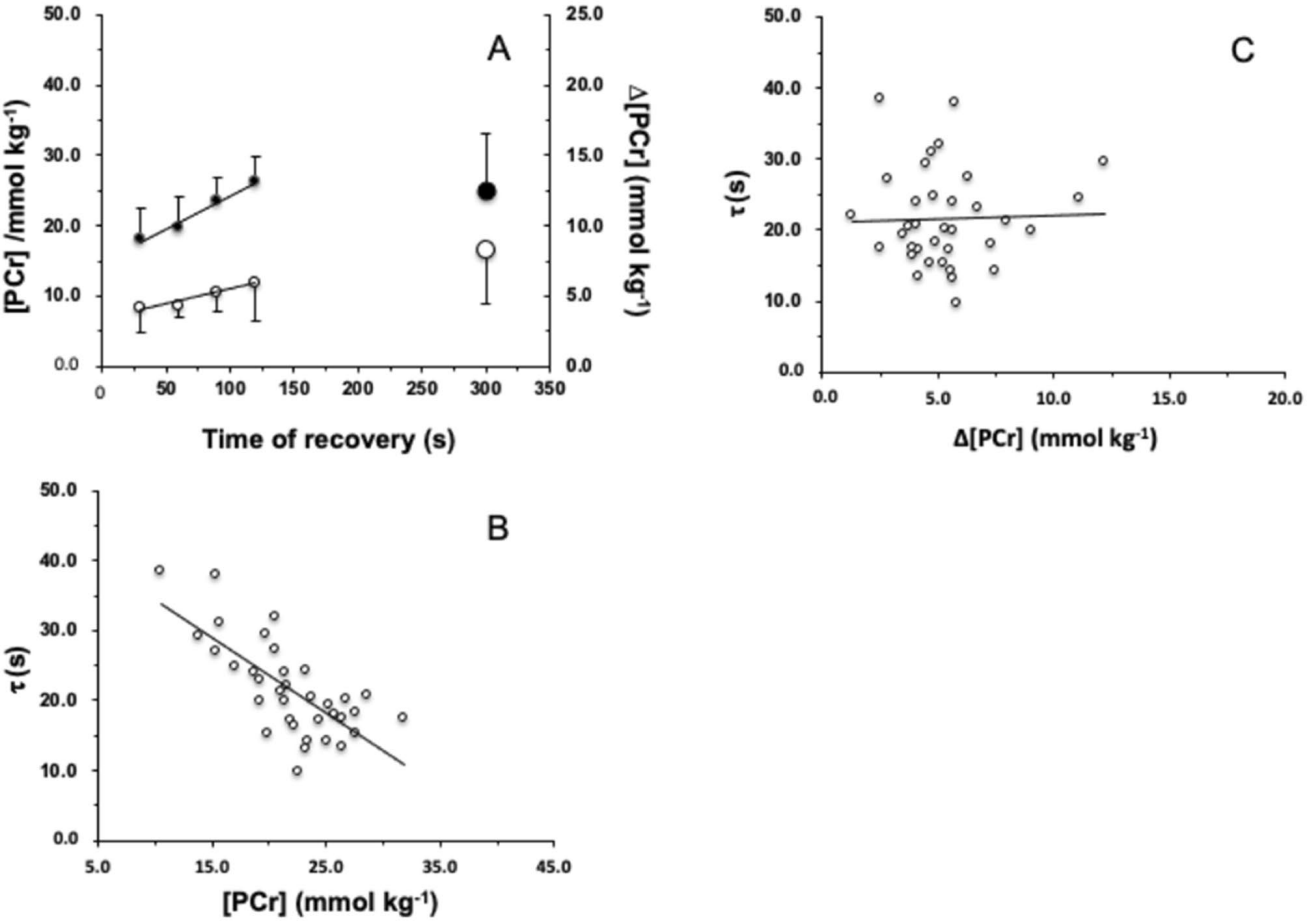
**A** Estimated [PCr] (full circles) and ∆[PCr] (open circles) prevailing before the onset of square-wave exercise as a function of the time of recovery. Big circles (at time of recovery of 300 s) refer to the control conditions. [PCr] at rest and after 300 s of recovery was assumed equal to 25 mmol kg^−1^ of fresh muscle; ∆[PCr] values at rest and after a complete recovery were used to obtain a single mean value: **B** *τ* as a function of [PCr] prevailing at the end of recovery before the onset square-wave transitions; **C** *τ* as a function of ∆[PCr] consumed during the transient phase of each square-wave transitions

In Fig. 4, the estimated [PCr] and ∆[PCr] are plotted as a function of the time of recovery preceding the exercise transitions. Both quantities increased as a function of a time of recovery up to 120 s ([PCr] = 0.094 time + 14.9, *r*^2^ = 0.979; ∆[PCr] = 0.021 time + 3.33, *r*^2^ = 0.94). The *τ* of the primary phase of $\dot{V}{\text{O}}_{{2{\text{A}}}}$ kinetics turned out to be a decreasing function of the [PCr] (*τ* = − 1.07 [PCr] + 44.9, *r*^2^ = 0.513, *P* < 0.01; *F* = 34.8). Conversely, it seemed not to be affected by ∆[PCr] (*τ* = 0.096 ∆[PCr] + 20.96, *r*^2^ = 0.001, *F* = 0.033). Given these results, one may also expect that the *τ* $\dot{V}{\text{O}}_{2}$ on-kinetics depends on the recovery time according to a first-order decaying function (Vinetti et al. 2017), as it is suggested in Fig. 3.

Therefore, these present calculations yield results that contrast with the experimental (Francescato et al. 2008) and predicted data (Korzeniewski and Zoladz 2004) and also with the predictions according to the model of Meyer (1988)

### $\dot{V}{\text{O}}_{{2{\text{A}}}}$ kinetics and energetic muscle state

The obtained data, although in contrast with those predicted (Korzeniewski and Zoladz 2004) or showed (Francescato et al. 2008), agree with the findings presented by other investigators who showed that the *τ* of the primary phase is significantly longer when the square-wave transient is imposed after 20 s of recovery following the abrupt interruption of moderate-intensity, constant rate exercise, so that exercise is resumed from an increased level of the metabolism (Bowen et al. 2011).

Several biochemical/enzymatic and thermodynamic modifications may explain the altered $\dot{V}{\text{O}}_{{2{\text{A}}}}$ of exercise starting from an elevated baseline metabolic rate. It has been proposed that mitochondrial oxidative rate conforms to Michaelis–Menten enzyme kinetics via the substrates involved in ATP resynthesis (Chance et al. 1981). In this case, [ADP] and [P_i_] would influence the rate of increase of $\dot{V}{\text{O}}_{{2{\text{A}}}}$. For instance, *K*_m_ for ADP depends upon [ATP] and [P_i_] (Wilson 1994). Therefore, when metabolic rate and mitochondrial respiration are elevated, the signal deriving from the phosphorylation potential ([ATP]/ [ADP] × [P_i_]) should be more prominent to elicit a given increase of $\dot{V}{\text{O}}_{{2{\text{A}}}}$. This speculation is further strengthened by the findings that oxidative phosphorylation is not linearly related to the free energy of ATP hydrolysis (∆*G*_ATP_) which is, in turn, affected by metabolite concentrations. This matter of fact would imply that more significant changes in [ADP] and [P_i_] would have been required for each increase of oxidative phosphorylation at elevated metabolic rates (Jeneson et al. 1995).

The reduced value of the starting ∆G_ATP_ at the onset of exercise would require a greater ATP synthesis. This mechanism would also predict a higher $\dot{V}{\text{O}}_{{2{\text{ss}}}}$ (and a more extensive functional gain, i.e., the net increment of $\dot{V}{\text{O}}_{{2{\text{A}}}}$ per unit of workload increase) for the same absolute workload. This in turn implies that $\dot{V}{\text{O}}_{{2{\text{A}}}}$ would take more time to attain $\dot{V}{\text{O}}_{{2{\text{ss}}}}$ with the progressive fall of ∆*G*_ATP_ as suggested by others (Bowen et al. 2011). However, this mechanism cannot be advocated as the possible explanation of the observed findings in the present investigation, since neither $\dot{V}{\text{O}}_{{2{\text{ss}}}}$ nor gains were affected by recovery time.

In contrast with the results obtained in this investigation, on one hand, starting from an elevated baseline metabolic rate did not decelerate the $\dot{V}{\text{O}}_{{2{\text{A}}}}$ kinetics during bouts of high-intensity exercise initiated from a $\dot{V}{\text{O}}_{2}$ level identical to $\dot{V}{\text{O}}_{{2{\text{ss}}}}$ of moderate-intensity exercise (DiMenna et al. 2010). On the other hand, the *τ* of the $\dot{V}{\text{O}}_{{2{\text{A}}}}$ kinetics investigated during high-intensity exercise transitions performed from unloaded pedaling was significantly longer than the present results (DiMenna et al. 2010). The slowed $\dot{V}{\text{O}}_{{2{\text{A}}}}$ kinetics may be the consequence of the more extensive recruitment of fast motor units at higher work rates than occurring at the onset of moderate-intensity exercise, as in the present case. The motor units progressively recruited at higher work rates are formed by IIX fibers characterized by lower mitochondrial content and P/O ratio, longer PCr splitting kinetics, and higher [PCr], and moreover, they rely more on the anaerobic glycolytic production of ATP. All these features have been directly related to a slower $\dot{V}{\text{O}}_{{2{\text{A}}}}$ kinetics (Korzeniewski and Zoladz 2004). For all these reasons, the results suggesting a slower response of the oxidative energy-yielding pathway obtained during rest-to high-intensity exercise transition cannot be directly compared with those obtained in the present investigation. They should be likely attributed to different neuromuscular, cellular, and biochemical mechanisms.

### $\dot{V}{\text{O}}_{{2{\text{A}}}}$ kinetics and circulatory dynamics

The results presented by Francescato and colleagues (Francescato et al. 2008) concern the *τ* of PCr splitting were obtained investigating the time course of PCr disappearance and synthesis using magnetic resonance spectroscopy applied on small muscle groups.

Our experiments were carried out in vivo on exercising humans. They considered only alveolar breath-by-breath O_2_ transfer and estimated the corresponding [PCr] absolute concentrations and changes. At the sudden onset of a constant-load exercise, $\dot{V}{\text{O}}_{{2{\text{A}}}}$ conforms to a two-component time course whose first small amplitude and rapid phase are mainly dictated by the rapid increase of pulmonary blood flow. Only the second one (metabolic or primary phase) is a reliable proxy of muscular O_2_ uptake. However, as it has been appropriately pointed out that (Francescato et al. 2013), between the muscle, i.e., the final utilization site of O_2_, and the lung, where O_2_ uptake is measured, at least two buffers exist. They are the cardiovascular system, since the dynamic response of convective O_2_ transport may distort alveolar O_2_ uptake and the interposition of the amount of O_2_ bound, before exercise transient, to the O_2_ venous stores. The effect of the O_2_ stored inside the body before the exercise transient, and already available to the muscles, consists in slowing down $\dot{V}{\text{O}}_{{2{\text{A}}}}$ kinetics, as the latter measures only the volume of O_2_ taken up from the environment and transferred across the alveolar-capillary membrane. Thus, on purely theoretical grounds, the slower alveolar $\dot{V}{\text{O}}_{{2{\text{A}}}}$ kinetics might be attributed, ceteris paribus, to different absolute values of O_2_ venous stores prevailing at the end of each period of recovery and/or to a kinetic dissociation between the rate of O_2_ muscular uptake and the cardiovascular dynamics in the different evaluated recovery conditions. However, it has also been demonstrated with in silico computational simulation that the high $\dot{Q}/\dot{V}{\text{O}}_{{2{\text{m}}}}$ prevailing in the condition of the shortest recovery time (30 s) would elicit the decrease of *τ* − $\dot{V}{\text{O}}_{{2{\text{A}}}}$ (Bowen et al. 2011). Therefore, the deceleration of $\dot{V}{\text{O}}_{{2{\text{A}}}}$ kinetics observed in coincidence with the shortest recovery time should likely be of muscular origin.

In summary, the results obtained in this investigation do not agree with the view that a decreased pre-exercise [PCr] brings about a substantial acceleration of aerobic metabolism in the muscle whose dynamic adjustments are described in exercising humans by $\dot{V}{\text{O}}_{{2{\text{A}}}}$ kinetics. Instead, $\dot{V}{\text{O}}_{{2{\text{A}}}}$ kinetics seems to be influenced by the pre-transition metabolic rate, implying that the request of ATP mitochondrial production rises in proportion to the metabolism of the muscle and the consequent changes of intracellular energetic status.

## Abbreviations

*A*: Amplitude of the mono-exponential response
ATP: Adenosine triphosphate
[ATP]: Adenosine triphosphate concentration
[ADP]: Adenosine diphosphate concentration
CI95: 95% Confidence intervals
∆*G*_ATP_: Free energy of ATP hydrolysis
∆[PCr]: Amount of PCr split during rest-to-work transition
DefO_2_: Oxygen deficit
*G*: Functional gain of $\dot{V}{\text{O}}_{{2{\text{A}}}}$ at steady state
GET: Gas exchange threshold
HR: Heart rate
[La]_b_: Blood lactate concentration
[Pi]: Inorganic muscle phosphate concentration
[PCr]: Phosphocreatine concentration
*τ* (tau): Time constant
*τ* [PCr]: Time constant of phosphocreatine splitting kinetic
*τ*_1_: Time constant of (Phase 1, or cardio-dynamic phase) of alveolar O_2_ kinetic
*τ*_2_: Time constant of (Phase 2, or primary phase) of alveolar O_2_ kinetic
$$\dot{V}{\text{O}}_{2}$$: Oxygen consumption
$\dot{V}{\text{O}}_{{2{\text{A}}}}$: Alveolar oxygen uptake
$\dot{V}{\text{O}}_{{2{\text{m}}}}$: Uptake at muscles levels
$\dot{V}{\text{O}}_{{2{\text{ss}}}}$: Steady-state oxygen uptake
$\dot{V}{\text{O}}_{{2{\text{peak}}}}$: Peak oxygen uptake
WR: Work rate
